# Correction: The structural, vibrational, and mechanical properties of jammed packings of deformable particles in three dimensions

**DOI:** 10.1039/d2sm90054h

**Published:** 2022-05-04

**Authors:** Dong Wang, John D. Treado, Arman Boromand, Blake Norwick, Michael P. Murrell, Mark D. Shattuck, Corey S. O'Hern

**Affiliations:** Department of Mechanical Engineering & Materials Science, Yale University New Haven Connecticut 06520 USA corey.ohern@yale.edu; Integrated Graduate Program in Physical and Engineering Biology, Yale University New Haven Connecticut 06520 USA; Department of Physics, Yale University New Haven Connecticut 06520 USA; Department of Biomedical Engineering, Yale University New Haven Connecticut 06520 USA; Systems Biology Institute, Yale University West Haven Connecticut, 06516 USA; Benjamin Levich Institute and Physics Department, The City College of New York New York New York 10031 USA; Department of Applied Physics, Yale University New Haven Connecticut 06520 USA

## Abstract

Correction for ‘The structural, vibrational, and mechanical properties of jammed packings of deformable particles in three dimensions’ by Dong Wang *et al.*, *Soft Matter*, 2021, **17**, 9901–9915, DOI: 10.1039/D1SM01228B.

The authors regret the error in eqn (3) and the subsequent errors in the surrounding paragraph. The correct text for eqn (3) and the surrounding paragraph is given below.

We calculate the Love stress tensor under periodic boundary condition^1^ using3
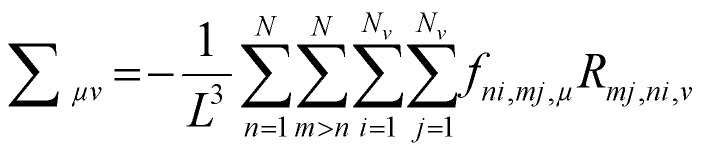
where *μ,ν = x,y,z, f*_*ni,mj,μ*_ is the *μ*th component of the force on vertex *i* belonging to particle *n* from vertex *j* belonging to particle *m, R*_*mj,ni,ν*_ is *ν*th component of the separation vector from the center of mass of particle *n* to the contact point between vertex *i* on particle *n* and vertex *j* on particle *m*. The pressure is defined as 
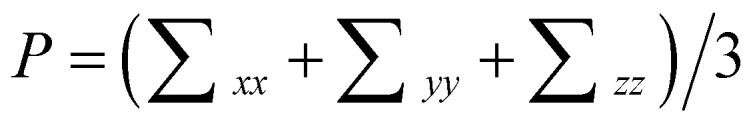
. We have verified that eqn (3) gives the same value for the pressure and shear stress compared to those obtained by calculating the change in the total potential energy with respect to changes in area and shear strain.

The Royal Society of Chemistry apologises for these errors and any consequent inconvenience to authors and readers.

## Supplementary Material
